# Evaluation and Management of Recurrent Atrial Flutter in Neonates

**DOI:** 10.3390/jcm14197126

**Published:** 2025-10-09

**Authors:** Nandini Aravindan, Peter R. A. Gaskin, Sudhir Vashist

**Affiliations:** 1 Department of Obstetrics & Gynecology, North Shore University Hospital & Long Island Jewish Medical Center, Manhasset, NY 11030, USA; naravindan@northwell.edu; 2 Division of Pediatric Cardiology, Department of Pediatrics, University of Maryland School of Medicine, Baltimore, MD 21201, USA; pgaskin@som.umaryland.edu

**Keywords:** neonatal arrhythmia, neonatal atrial flutter, recurrent neonatal atrial flutter

## Abstract

**Background**: Fetal tachyarrhythmias occur in less than 0.1% pregnancies, with atrial flutter accounting for one-third of cases. Atrial flutter results from a reentrant circuit within the atrium with atrial rates in fetal atrial flutter ranging from 300 to 540 beats per minute. The fetal atrial flutter is most often an isolated finding; however, it may also be associated with maternal diabetes, neonatal macrosomia, cardiac rhabdomyoma, maternal substance use, Turner syndrome, congenital heart disease, and the presence of accessory pathways. The majority of cases of atrial flutter in the neonatal period are isolated; however, only a few cases of recurrent atrial flutter have been described. **Methods**: This is a single-institution, retrospective chart review of neonates with recurrent atrial flutter. **Results**: Four neonates with recurrent atrial flutter were identified, each linked either to a correctable trigger or to an underlying substrate, guiding individualized therapy. When no clear trigger was present, antiarrhythmic medication was required. **Conclusions**: These cases highlight the importance of the recognition of potential triggers of recurrent neonatal atrial flutter, tailoring therapy accordingly and considering antiarrhythmic agents when necessary.

## 1. Introduction

Fetal tachyarrhythmias occur in less than 0.1% of pregnancies [1]. The most common cause is atrioventricular reentrant supraventricular tachycardia mediated by an accessory pathway. Atrial flutter is responsible for one-third of fetal tachyarrhythmias and is caused by a reentrant circuit within the atrium [2]. Atrial rates in fetal atrial flutter range between 300 and 540 beats per minute [3]. Fetal atrial flutter is most often an isolated finding; however, it may also be associated with maternal diabetes, neonatal macrosomia [4], cardiac rhabdomyoma [5], maternal substance use [6], Turner syndrome [7], congenital heart defect (CHD) [3], and the presence of accessory pathways [8,9]. 

Atrial flutter can be managed medically in utero with digoxin, sotalol, or flecainide [2]. Sotalol is considered first-line therapy due to its high efficacy [2]. In neonates with atrial flutter, cardioversion or transesophageal atrial overdrive pacing is the treatment of choice with a rare need for ongoing medical management with no further recurrences [2,10]. 

The majority of cases of atrial flutter in the neonatal period are isolated. Few cases of recurrent atrial flutter have been described. Texter et al. [11] reviewed cases of atrial flutter with most recurrences requiring antiarrhythmic medication. Multiple cardioversions and rarely catheter ablation have also been described as a treatment option [12,13,14,15]. 

Our case series reports four cases in which atrial flutter recurred in the neonatal period. We hypothesize that the recurrences were due to either triggers or underlying substrates. In these cases, the underlying etiology was identified and addressed. If no obvious trigger or substrate is identified, antiarrhythmic medication may be required. 

## 2. Methods

This single-center, retrospective case series was conducted using institutional medical records from the University of Maryland Medical Center between January 2019 and December 2023. Cases were identified as those with an electrocardiographic diagnosis of atrial flutter, who experienced a recurrence of atrial flutter, and who were age <28 days at initial occurrence. Exclusion criteria included isolated episodes of atrial flutter as diagnosed by electrocardiogram and age >28 days of life at the time of the first episode. Clinical data were extracted from the electronic medical records, including perinatal history, ECG findings, echocardiographic results, telemetry, treatment modalities, and short-term follow-up clinic appointments. This study was exempt from review by the institutional review board under category 4.

### 2.1. Case Reports

#### 2.1.1. Case 1

A term, female, large-for-gestational-age newborn was born via Cesarean section to a diabetic mother on insulin therapy. The neonate was hypoglycemic after birth, requiring neonatal ICU care. On day 2 of life, the neonate was noted to be tachycardic. Electrocardiogram showed atrial flutter that responded to synchronized cardioversion. The atrial flutter recurred on day three. Telemetry showed premature atrial contractions triggering atrial flutter. Chest X-ray revealed an umbilical venous catheter that had migrated deep in the right atrium. The catheter was readjusted to the inferior vena cava–right atrium junction, and a second synchronized cardioversion was performed with immediate success. No further recurrences of premature atrial contractions or atrial flutter were noted during the rest of the hospital stay or during outpatient follow-up. 

#### 2.1.2. Case 2

**Figure 1:**
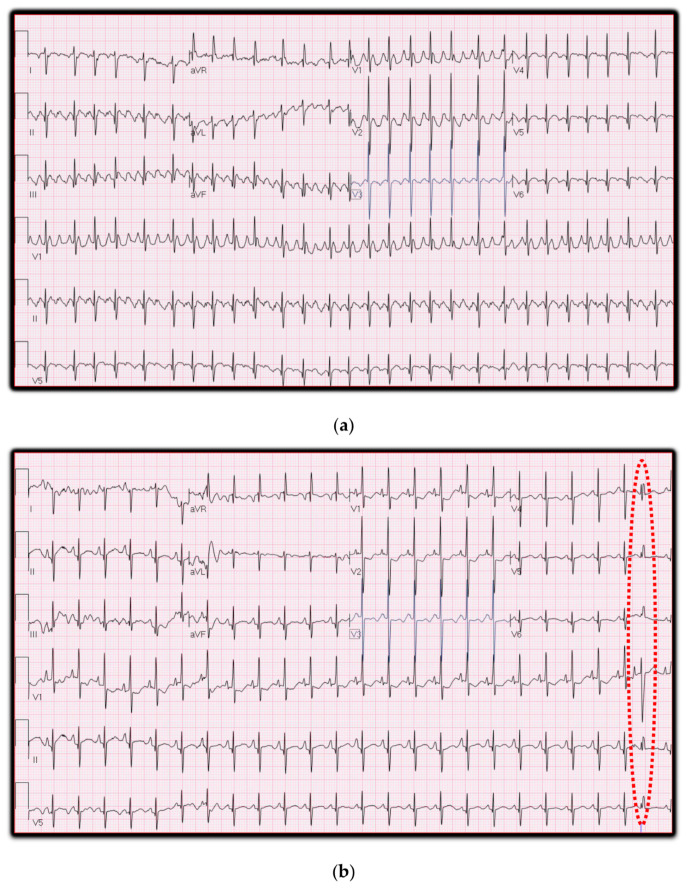
Electrocardiogram showing atrial flutter (**top panel**) and premature atrial contractions during sinus rhythm (**bottom**). (**a**): Atrial Flutter at 440 beats per minute (BPM) and mostly 2:1 atrio ventricular (AV) Conduction (**b**): ECG performed in Sinus Rhythm for the same patient showing premature atrial contraction (PAC) with aberrant conduction (highlighted with red dotted oval).

A term female infant born at 39-week gestation via Cesarean section was transferred to our institution on day 1 of life. The fetus was noted to be tachycardic to 220–230 beats per minute just prior to delivery. After delivery, the electrocardiogram confirmed atrial flutter at 440 beats per minute with mostly 2:1 atrioventricular conduction (Figure 1a). The neonate was cardioverted multiple times using synchronized cardioversion with success on the third attempt with 1 J/Kg of energy. Atrial flutter recurred on multiple occasions after cardioversion, and these episodes self-converted to sinus rhythm. The patient had premature atrial contractions while in sinus rhythm (Figure 1b), and a review of telemetry suggested that these were the trigger for atrial flutter episodes. The echocardiogram revealed right atrial dilation. Propranolol was initiated to suppress premature atrial contractions. The patient was discharged on propranolol, and right atrial dilation resolved during follow-up. Propranolol was discontinued at around one year of age with no further recurrences during follow-up. 

#### 2.1.3. Case 3

A female infant born at 35-week gestation via Cesarean section was transferred to our institution on day 0 of life. Fetal supraventricular tachycardia was incidentally noted on prenatal ultrasound two days prior. Pregnancy was otherwise uncomplicated. Soon after delivery, the neonate was noted to have atrial flutter with ventricular rates of 260 beats per minute. The echocardiogram at the outside hospital showed normal cardiac structure and function. Episodes of atrial flutter were recurrent but self-resolving. Propranolol was initiated; however, as episodes of atrial flutter persisted despite propranolol, sotalol was added to the regimen and slowly up-titrated. The neonate did not have any further episodes on this combined therapy. Antiarrhythmics were discontinued at the end of the first year of life with no further recurrences during follow-up.

#### 2.1.4. Case 4

A term male newborn with a prenatal diagnosis of truncus arteriosus type II developed recurrent episodes of atrial flutter after the repair of truncus arteriosus. The repair involved the Gortex patch closure of the ventricular septal defect, main pulmonary artery plasty, patch augmentation of the aorta, partial closure of the atrial septal defect, and placement of a right ventricle (RV) to the pulmonary artery conduit. The bypass time was 375 min, and the cross-clamp time was 118 min. Given significant RV dysfunction and hypoxia coming off bypass, the conduit was revised, and the patient required extra-corporal membrane oxygenator therapy (ECMO) and inotropic support.

While ECMO support was being weaned, this patient developed recurrent episodes of atrial ectopy and then atrial flutter. Many of these episodes were associated with hemodynamic instability, and temporary atrial pacing wires placed at the time of surgery were used to successfully overdrive pace the neonate out of atrial flutter. However, given the recurrent nature and associated hemodynamic instability, amiodarone was initially started, and later flecainide was added for optimal arrhythmia control. As many episodes developed when magnesium levels were below 1.8 mg/dL, magnesium supplementation was added. At 4 ½ months of age, a pseudo-aneurysm at mid-conduit and distal conduit stenosis was identified; conduit revision was performed with a 12 mm Hancock valved conduit. Atrial flutter recurred in the post-operative period, and antiarrhythmic doses were titrated to control the arrhythmia.

This patient required g-tube and tracheostomy placement and was mechanically ventilated. There were no arrhythmias in the outpatient setting after discharge at 6 months of age. Antiarrhythmic medications were gradually weaned off at age 3 years with no further recurrence during follow-up.

## 3. Discussion

**Figure 2:**
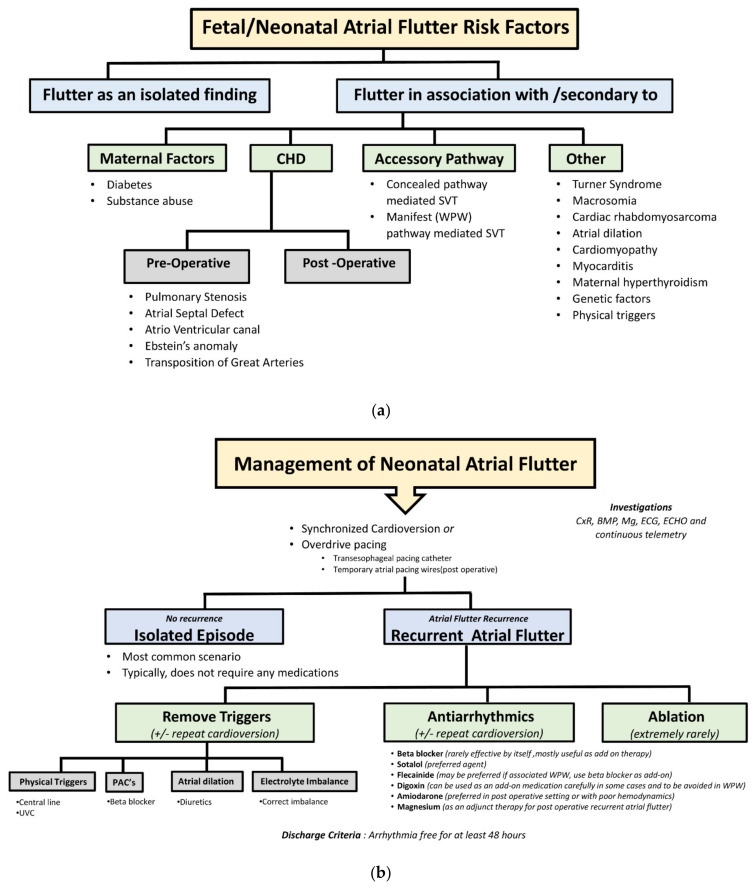
Atrial flutter management. (**a**): Risk Factors for Fetal/Neonatal Atrial Flutter, CHD: Congenital Heart Defect, WPW: Wolff-Parkinson White Syndrome, SVT: Supra Ventricular Tachycardia; (**b**): Management of Neonatal Atrial Flutter. UVC: Umbilical Venous Catheter, WPW: Wolff-Parkinson White Syndrome, PAC: Premature Atrial Contraction, BMP: Basic Metabolic Panel, Mg: Magnesium.

Atrial flutter comprises approximately one-third of fetal tachyarrhythmias [2]. If fetal atrial flutter does not resolve spontaneously, it may have an adverse outcome on fetal wellbeing, potentially resulting in hydrops fetalis, heart failure, and fetal demise if severe and uncontrolled [9,10,11,13]. Fetal and neonatal atrial flutters mostly occur in isolation but can be secondary to diverse etiologies (Figure 2a). These underlying causes may be of maternal origin, including maternal diabetes [4] or substance use [6]; in association with CHD [3]; secondary to substrates created from the surgical repairs of CHD; or in association with accessory pathways [8,9]. 

The treatment of choice for a newborn with atrial flutter is cardioversion or overdrive pacing via transesophageal catheter or temporary atrial pacing wires (Figure 2b). These therapies are typically curative with no recurrences, and ongoing medical management is not required. In cases of atrial flutter as an isolated episode, follow-up for up to one year of life is reasonable to assess for accessory pathways as they could be present in approximately 10% of patients with a history of neonatal atrial flutter and may result in supraventricular tachycardia [3,8]. 

There is a paucity of data regarding the etiology and management of recurrent neonatal flutter. In one of the largest series of neonatal atrial flutter by Texter et al. [11], the recurrence rate of atrial flutter was 12%, and all patients with recurrence had additional arrhythmias, most requiring antiarrhythmic medication. Catheter ablation has been employed as a last-resort therapy in cases where medical management fails and atrial flutter persists, particularly when complicated by tachycardia-induced cardiomyopathy [15]. In this brief report, we describe four neonates with recurrent atrial flutter, focusing on the causes of recurrences and tailoring therapy according to the triggers identified (Figure 2b). If a physical trigger such as an umbilical venous catheter is identified, its placement must be modified. If a premature atrial contraction is identified as the trigger on electrocardiogram, beta blockers alone may be effective. In the presence of additional arrhythmias such as supraventricular tachycardia, beta blockers or additional antiarrhythmic medications may be needed. If atrial dilation is noted in association with atrioventricular valve regurgitation or ventricular dysfunction, diuretics may be effective in reducing the atrial size. If post-surgical inflammation and scarring are the cause, it may take some time for these triggers to abate. Inotropic agents or other therapies for optimizing ventricular function along with antiarrhythmics may be required in the interim. Electrolytes must be closely monitored and corrected if imbalances are found. If there is no identifiable trigger, an antiarrhythmic medication may need to be initiated. Sotalol is the most effective agent and remains the first line for fetal treatment, especially in the presence of fetal hydrops, as it effectively converts 50–80% fetuses without mortality [2]. However, additional medications such as flecainide or digoxin may be required. Rare cases of those with recurrent neonatal atrial flutter who fail these therapies may need amiodarone, with rare case reports describing ablation as a treatment strategy in refractory cases [8,15]. However, given the risk vs. benefit ratio of catheter ablation in the neonatal age group, this treatment modality is used extremely rarely and reserved as a last-resort treatment option for refractory recurrent atrial flutter that is associated with hemodynamic compromise or tachycardia-induced cardiomyopathy. 

## 4. Limitations

This study has several limitations. It is a retrospective short case series that was conducted at a single tertiary care center. Only four neonates with recurrent atrial flutter were identified, which limits the ability to draw broader conclusions about incidence, etiology, and optimal management strategies. In addition, as a tertiary referral center, these cases may represent the more severe and complex end of the spectrum. The follow-up data was also limited to the first few years of life. A longer follow-up is needed to assess the recurrence risk beyond infancy, the development of supraventricular tachycardia mediated by accessory pathways, and the long-term outcomes of antiarrhythmic therapy. Future larger multicenter studies with extended follow-up are warranted to better guide the management of recurrent neonatal atrial flutter and characterize outcomes.

## 5. Conclusions

Neonatal atrial flutter generally carries an excellent prognosis, and once converted to sinus rhythm, recurrence is uncommon with no need for ongoing medical therapy. In the uncommon scenario of the recurrence of atrial flutter, it is critical to identify and address potential triggers and to initiate antiarrhythmic therapy when indicated. This case series identified four neonates with recurrent atrial flutter who required tailored management approaches. A brief follow-up is reasonable to assess for the concomitant possibility of an accessory pathway that may predispose patients to supraventricular tachycardia in the future [8].

## Data Availability

Data is contained within the article.
